# *Cystobacter fuscus* HM-E: a novel biocontrol agent against cotton Verticillium wilt

**DOI:** 10.3389/fmicb.2025.1555523

**Published:** 2025-03-12

**Authors:** Jian Han, Meili Shi, Xinyu Dou, Wen Pan, Deying Ma, Ming Luo, Benzhong Fu

**Affiliations:** ^1^Department of Plant Pathology, College of Agronomy, Xinjiang Agricultural University, Urumqi, China; ^2^Key Laboratory of Prevention and Control of Invasive Alien Species in Agriculture and Forestry of the North-western Desert Oasis (Co-construction by Ministry and Province), Ministry of Agriculture and Rural Affairs, Urumqi, China; ^3^Engineering Research Centre of Cotton, Ministry of Education, Urumqi, China

**Keywords:** *Verticillium dahlia*, cotton Verticillium wilt, *Cystobacter fuscus*, myxobacteria, biocontrol, *Protaetia brevitarsis* frass, antifungal mechanisms

## Abstract

Verticillium wilt of cotton, caused by *Verticillium dahliae*, is one of the most devastating soilborne fungal diseases in cotton production, urgently demanding the development of effective control measures. Myxobacteria, a group of higher prokaryotes exhibiting multicellular social behaviors, possess predatory activity against plant pathogenic fungi and bacteria, giving them unique potential for application in plant disease biocontrol. In this study, based on a previously myxobacterial strain collection, a myxobacterial strain, HM-E, exhibiting broad-spectrum antifungal activity was screened. Through morphological observation, physiological and biochemical characterization, and multi-locus sequence analysis, this strain was identified as *Cystobacter fuscus* HM-E. *C. fuscus* HM-E not only significantly lysed *V. dahliae* hyphae but also inhibited its spore germination. Both its cell-free fermentation filtrate and volatile metabolites exhibited certain antifungal activity. Greenhouse pot assays showed that the fermentation broth of *C. fuscus* HM-E had a control efficacy of only 23.01% against cotton Verticillium wilt, whereas the solid agent formulated with white star flower chafer (*Protaetia brevitarsis*) frass achieved a significantly higher control efficacy of 70.90%, and the myxobacterial solid agent also significantly promoted cotton seedling growth. Furthermore, the crude extracts concentrated using macroporous resin and acid precipitation showed no antifungal activity against *V. dahliae*, whereas the crude protein obtained by ammonium sulfate precipitation disrupted not only the cell wall and cell membrane of *V. dahliae* hyphae, induced intracellular reactive oxygen species (ROS) burst but also lysed spores and inhibited spore germ tube elongation. Enzyme substrate profile assays indicated that several peptidases, lipases, and glycoside hydrolases secreted by *C. fuscus* HM-E might play important roles in its antifungal process and are potential biocontrol factors. This study suggests *C. fuscus* HM-E, as a novel biocontrol agent, has great potential for application in the combating of cotton Verticillium wilt.

## 1 Introduction

China is the world’s largest cotton producer and consumer. Xinjiang, the largest cotton production base in China, accounted for over 90% of the national planting area in 2023, with a cotton output of 5.112 million tons (National Bureau of Statistics of China). Xinjiang is also a globally important production base for extra-long staple cotton and colored cotton, and the high-quality cotton from some of its cotton-producing areas has become the preferred choice for several mid-to-high-end cotton textile enterprises. Therefore, cotton production in Xinjiang is crucial for local economic development.

Verticillium wilt of cotton, caused by *V. dahliae*, is one of the major constraints to high-quality and high-yield cotton production in Xinjiang, often referred to as “cotton cancer,” severely impacting cotton yield and fiber quality (Umer et al., 2023). In recent years, the incidence of cotton Verticillium wilt has been increasingly severe due to factors such as inter-regional transfer of cotton varieties, straw return to the field, continuous cropping, and agricultural management practices (Zhang et al., 2023a). Controlling cotton Verticillium wilt is challenging, and no specific curative agents are available to date. Current main strategies for controlling the disease include crop rotation, resistant variety breeding, chemical control, and biological control. However, due to the rapid mutation rate, broad host range, and high pathogenicity of *V. dahliae*, obtaining resistant varieties is difficult (Aini et al., 2022). Although chemical pesticides, represented by carbendazim, play an important role in the chemical control of cotton Verticillium wilt (Hu et al., 2024), over-reliance and abuse of chemical pesticides lead to a series of problems, including enhanced pathogen resistance, environmental pollution, and disruption of beneficial soil microorganisms, seriously affecting the green and sustainable development of the cotton industry (Rani et al., 2021). Therefore, there is an urgent need to explore safe and efficient biological control measures to ensure the sustainable and healthy development of the cotton industry.

Biological control technologies have received increasing attention due to their persistent control effects, absence of residues, high specificity against target pathogens, and compatibility with other control measures (Lahlali et al., 2022). In recent years, numerous studies have screened antagonistic microorganisms from soil, phyllosphere, and endosphere, encompassing various biocontrol microbial groups, including bacteria, actinomycetes, fungi, and fungal viruses. Examples of bacterial biocontrol agents include *Bacillus subtilis* (Zhao et al., 2022), *B. amyloliquefaciens* (Liu et al., 2023), *Pseudomonas fluorescens* (Otsu et al., 2004), and *Burkholderia* sp. (Tian et al., 2022). Well-studied antagonistic fungi mainly include *Trichoderma* sp. (Carrero-Carrón et al., 2016), *Talaromyces flavus* (McLaren et al., 1986), and *Chaetomium* sp. (Zhang et al., 2021). Among actinomycetes, *Streptomyces* sp. (Xue et al., 2016), has been most extensively studied and applied. The mechanisms of action of these strains include antagonism, mycoparasitism, nutrient or niche competition, and induction of plant systemic resistance. However, research and application of microbial predation for the biological control of cotton Verticillium wilt are still scarce.

Myxobacteria, a group of higher prokaryotes exhibiting social and predatory behaviors, are widely distributed in soil and are indigenous soil inhabitants (Zhou et al., 2014). Studies have shown that myxobacteria not only actively prey on other microorganisms through “wolfpack” predation and gliding motility but also differentiate into highly resistant myxospores, possessing strong environmental adaptability. Furthermore, they can produce a rich diversity of secondary metabolites and hydrolytic enzymes (Konovalova et al., 2010; Schaberle et al., 2014; Ye et al., 2020b). Moreover, myxobacteria occupy the top trophic level in the soil microbial food web, and their predation on soilborne pathogens directly affects the soil micro-ecological environment, playing a crucial role in maintaining soil microbial balance and plant health (Marshall and Whitworth, 2019). These characteristics confer unique biocontrol advantages to myxobacteria, making them recognized as a novel type of biocontrol microorganism.

Recent studies have revealed that myxobacterial strains from different genera exhibit antibacterial activity against various types of plant pathogens, demonstrating potential application value in plant disease biocontrol, especially showing significant control effects against some soilborne diseases. Bull et al. (2002) demonstrated that six isolated *Myxococcus* strains exhibited lytic and predatory effects against eight soilborne plant pathogenic fungi and could control lettuce drop caused by *Sclerotinia minor*. Dahm et al. (2015) isolated 30 myxobacterial strains from forest soil and tested their biocontrol properties against major fungal pathogens of pine seedlings. Pot experiments showed that some of these myxobacteria could protect seedlings from *Rhizoctonia solani* infection and also demonstrated their good colonization ability in potting soil. Li et al. (2017) found that the myxobacterium *Corallococcus* sp. EGB exhibited broad-spectrum predatory activity against various plant pathogenic fungi, such as *Fusarium oxysporum*, and bacteria. Pot and field efficacy tests showed that the solid agent prepared with strain EGB exhibited good biocontrol effects against cucumber Fusarium wilt and was superior to chemical treatments, significantly increasing crop yield.

Our laboratory has been focusing on isolating and screening myxobacterial strains from the unique geographical and ecological environment of Xinjiang that can effectively control cotton Verticillium wilt and be applied in Xinjiang cotton production areas. This study investigated a myxobacterial strain, HM-E, isolated from cotton field soil in Moyu County, Xinjiang, and found that it exhibited significant antifungal activity against *V. dahliae*. This study identified the myxobacterium, determined its biocontrol ability, and conducted an in-depth exploration of its biocontrol mechanisms.

## 2 Materials and methods

### 2.1 Strains, media, and plant material

The seven plant pathogenic fungi used in this study, *V. dahliae* (Vd, KC282468) and *F. oxysporum* f. sp. *vasinfectum* (Fov, KR071660) were kindly provided by Dr. Aixing Gu from Xinjiang Agricultural University, *Alternaria tenuissima* (At, OM884060), *Fusarium culmorum* (Fc, OP315277) and *Valsa mali* var. *pyri* (Vp, KY942187) were kindly provided by Dr. Lili Wang from Xinjiang Agricultural University, *Fusarium verticillioides* (Fv, OR105502) and *R. solani* (Rs, HF912170) were kindly provided by Dr. Qingyuan Guo from Xinjiang Agricultural University, and one plant pathogenic bacterium, *Erwinia amylovora* E.a001 (Ea) was isolated, identified, and preserved by our laboratory from pear tree branches infected with fire blight in a pear orchard in Korla City, Xinjiang (Lü et al., 2023), were all preserved in the Agricultural Microbiology and Biotechnology Laboratory of the College of Agriculture, Xinjiang Agricultural University.

Myxobacteria were isolated using WCX agar medium (Water Calcium and Cycloheximide) (Lee and Reichenbach, 2006): CaCl₂·2H₂O 1 g/L, agar powder 15 g/L, pH 7.2. After sterilization, cycloheximide was added to a final concentration of 25 μg/mL.

Strain HM-E was routinely cultured in LBS medium(Nair et al., 2019): soluble starch 7 g/L, casitone 1 g/L, yeast extract 5 g/L, MgSO₄·7H₂O 1 g/L, pH 7.2. The VY/2 medium used for plate growth inhibition assays consisted of: yeast 5 g/L, CaCl₂·2H₂O 1 g/L, agar powder 15 g/L, pH 7.2.

Potato Dextrose Agar (PDA) medium (potato 200 g/L, glucose 20 g/L, agar powder 15 g/L, natural pH) and Czapek-Dox medium (NaNO₃ 3 g/L, K₂HPO₄ 1 g/L, MgSO₄·7H₂O 0.5 g/L, KCl 0.5 g/L, FeSO₄ 0.02 g/L, sucrose 30 g/L, natural pH) were used for culturing and sporulation of the pathogenic fungi (Zhang et al., 2015b). Nutrient Agar (NA) (10 g/L peptone, 5 g/L NaCl, 3 g/L beef extract, pH 7.2) was used of the Ea culture.

The cotton cultivar “Xinluzao 18” (Verticillium wilt resistant) (Xie et al., 2005) used for biocontrol efficacy assays, was provided by Dr. Dawei Zhang of the Institute of Cash Crops, Xinjiang Academy of Agricultural Sciences.

### 2.2 Isolation and purification of strain HM-E

Soil samples were collected from Verticillium wilt-infected cotton fields in Moyu County, Hotan Prefecture, Xinjiang Uygur Autonomous Region, China (longitude 79°31′–79°34′E, latitude 37°11′–37°21′N, elevation 1,200–1,300 m). Approximately 200 g of soil was collected from the upper 5–10 cm layer, air-dried naturally, and stored at room temperature.

The isolation of myxobacteria followed the method described by Yi et al. (2021). Briefly, an aseptic inoculation loop was dipped into an *Ea* bacterial suspension (OD_600_ = 1.0) and streaked onto the surface of WCX medium in a four-square grid pattern.

**Figure 1:**
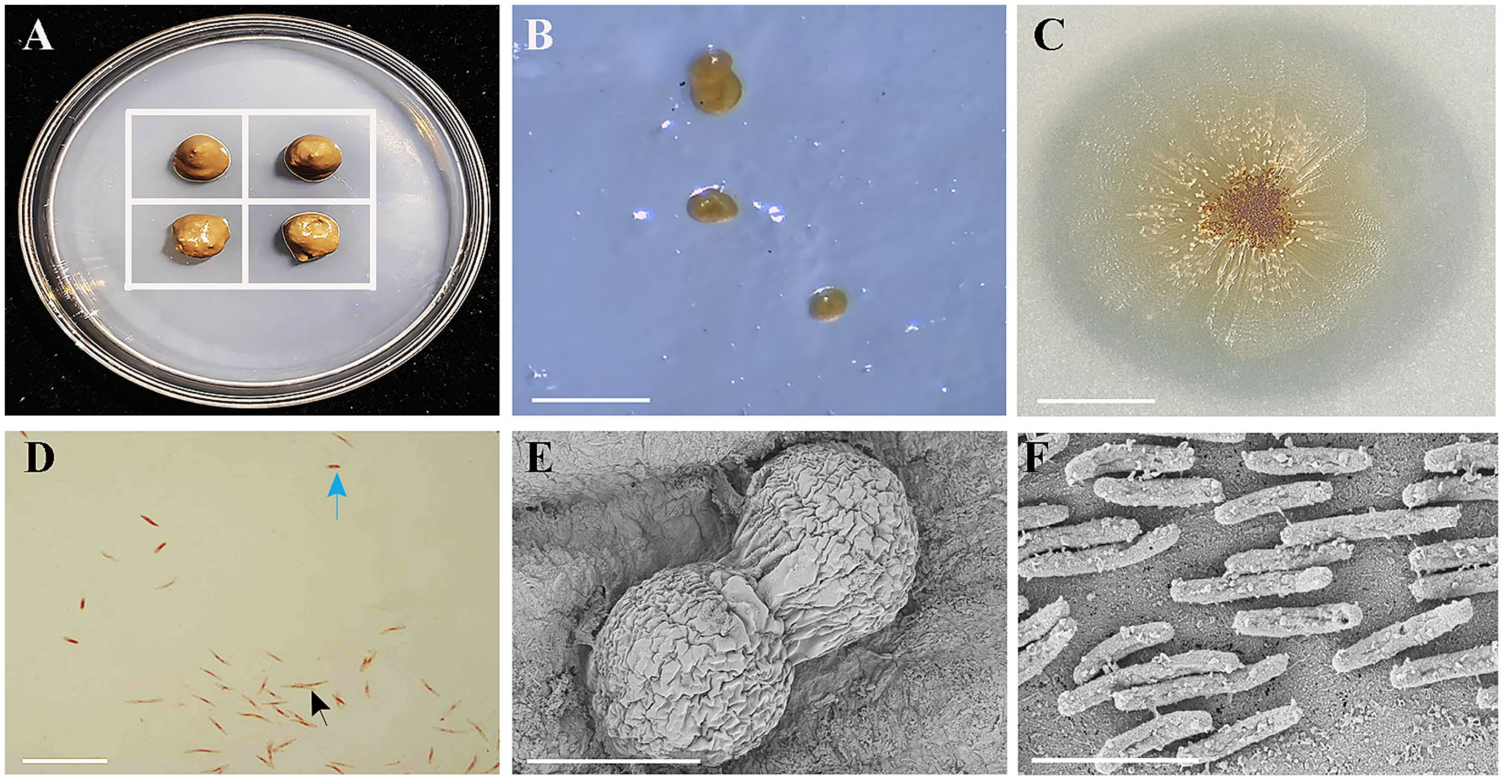
Isolation, colony morphology on VY/2 agar, Gram staining, and scanning electron microscopy (SEM) of strain HM-E. **(A)** Schematic diagram of the predatory bacteria induction method. The white grid lines represent the inoculated *Ea*. **(B)** Fruiting body formation of strain HM-E cultured on WCX medium for 5 days. Scale bar = 30 μm. **(C)** Colony morphology of strain HM-E on VY/2 agar. Scale bar = 3 mm. **(D)** Morphology of vegetative cells and myxospores of strain HM-E under light microscopy after Gram staining. Black arrows indicate vegetative cells, and blue arrows indicate myxospore. Scale bar = 15 μm. **(E)** SEM micrograph of fruiting bodies of strain HM-E. Scale bar = 60 μm. **(F)** SEM micrograph of vegetative cells of strain HM-E. Scale bar = 4 μm.

The soil samples treated with cycloheximide (100 μg/mL) were placed in the center of each square grid (Figure 1A) and incubated at 30°C for 72 h. The formation of fruiting bodies was observed under a stereomicroscope (SM7, Micro-Optic Instrument Group Co., Ltd.). Single fruiting bodies were picked with a sterile fine needle tip and transferred onto VY/2 medium for incubation at 30°C. After repeated purification to obtain pure cultures, the bacterial film was scraped off, suspended in 20% glycerol, and stored at −80°C for further use.

### 2.3 Morphological observation of strain HM-E

The fruiting body morphology of strain HM-E on WCX medium was observed and recorded using a stereomicroscope (SM7, Motic China Group Co., Ltd.). A pure culture of strain HM-E was inoculated onto VY/2 medium and incubated for 5 days to examine colony morphology. After Gram staining, the vegetative cells and myxospores of strain HM-E were observed under bright-field illumination using a fluorescence microscope (Ni-U, Nikon). Additionally, strain HM-E was fixed with 2.5% glutaraldehyde and sent to the Experimental Center of the Xinjiang Institute of Ecology and Geography, Chinese Academy of Sciences, for scanning electron microscopy (SEM, SUPRA55VP, Zeiss) to examine the fruiting body structure and cellular morphology.

### 2.4 Growth conditions and physiological and biochemical characteristics of strain HM-E

To determine optimal growth conditions, the effects of pH (5, 6, 7, 8, 9, and 10), temperature (16°C, 23°C, 30°C, 35°C, and 40°C), and NaCl concentration (0, 0.5, 1.0, 1.5, and 2.0%) on LBS medium were assessed, Each treatment was performed in triplicate. Cell dry weight was measured to evaluate growth performance (Zhang et al., 2015a).

Biochemical characterization of strain HM-E was conducted using a Microbiochemical Identification Kit (Haibo Biotechnology Co., Ltd., Qingdao, China). Antibiotic susceptibility testing was performed using antibiotic discs (Changde Bikeman Biotechnology Co., Ltd., China) following the recommended criteria of the Clinical Laboratory Standards Institute (CLSI, 2011). The standard *Escherichia coli* strain ATCC 25923 was used as a control. Each treatment was performed in triplicate. The results were compared with previously reported *Cystobacter* strains (Awal et al., 2017).

### 2.5 Molecular identification of strain HM-E

For bacterial DNA isolation, strain HM-E was cultured in 3 mL of LBS medium at 30°C with shaking at 180 rpm for 2 days. Cells were harvested by centrifugation at 10,000 × *g* for 2 min. Total DNA was extracted using the TIANamp Bacteria DNA Kit (TIANGEN Biotech (Beijing) Co., Ltd., China). The 16S rRNA gene was amplified using the universal primers 27F (5′-AGAGTTTGATCCTGGCTCAG-3′) and 1492R (5′-TACGGCTACCTTGTTACGACTT-3′) (Kumar et al., 2017). The *lepA* gene (leader peptidase GTP binding membrane protein) was amplified using the primers BAUP1 (5′-CATCGCCCA CATCGAYCAYGGNAA-3′) and BIDN1 (5′-CATGTGCAGCAGGC CNARRAANCC-3′) (Stackebrandt et al., 2005). The *gyrB* gene (DNA gyrase, subunit B) was amplified using the primers *gyrB*F (5′-GCGGA AGCGGCCNGSNATGTA-3′) and *gyrB*R (5′-CCGTCCACGTCGG CRTCNGYCAT-3′) (Santos and Ochman, 2004). The PCR products were sequenced using Sanger sequencing (Sangon Biotech (Shanghai) Co., Ltd., China). The obtained sequences were aligned against the NCBI database using BLAST. Phylogenetic trees based on the 16S rRNA, *lepA*, and *gyrB* gene sequences were constructed using the neighbor-joining method in MEGA 11.0.

### 2.6 Antifungal activity assay of strain HM-E

After activating strain HM-E on VY/2 plates for 5 days, a sufficient amount of bacterial film was scraped and inoculated into 3 mL of LBS broth. After incubation at 30°C and 180 rpm for 2 days, the culture was transferred to 200 mL of LBS broth and incubated at 30°C and 180 rpm for 3 days to obtain the fermentation broth of strain HM-E. The fermentation broth was centrifuged at 12,000 × *g* for 5 min to collect the cells, which were washed three times with sterile water. The aggregated myxobacterial cells were thoroughly dispersed and resuspended in sterile water to prepare a cell suspension with an OD₆₀₀ of 2.0. Mycelial plugs (*d* = 5 mm) of the seven plant pathogenic fungi described in Section 2.1 were inoculated in the center of VY/2 agar plates and incubated at 28°C until the colony diameter reached approximately 1.0 cm. Then, 20 μL of the HM-E cell suspension was streaked approximately 2 cm away from the edge of the fungal colony. Plates inoculated with the fungal pathogens alone served as controls. Each treatment was performed in triplicate. After 7 days of co-cultivation, the plates were photographed, and the diameters of the fungal colonies were measured (Dou et al., 2023). Furthermore, co-cultures of strain HM-E and *V. dahliae* on VY/2 plates were visualized using scanning electron microscopy (SEM, SUPRA55 VP, Zeiss).

### 2.7 *In vivo* biocontrol assay of strain HM-E against cotton Verticillium wilt

#### 2.7.1 Preparation of HM-E fermentation broth, solid agent, and *Verticillium dahlia* spore suspension

Strain HM-E was inoculated into 3 mL of LBS broth and incubated at 30°C and 180 rpm for 2 days. Then, the culture was transferred to 200 mL of LBS broth (in 500 mL Erlenmeyer flasks containing 300 glass beads (*d* = 3 mm) to promote dispersed growth) and incubated at 30°C and 180 rpm for 3 days to obtain the HM-E fermentation broth, which was then diluted with sterile water to an OD₆₀₀ of 1.0 for use.

White star flower chafer frass (provided by Golden Insect Biotechnology Co., Ltd) (sterilized at 121°C, 0.1 Mpa, 30 min) was used as the solid fermentation substrate. The myxobacterial fermentation broth (OD₆₀₀ = 1.0) was inoculated at a ratio of 50 mL/kg of sterile frass, thoroughly mixed, and adjusted to a moisture content of 60% with sterile water. The mixture was incubated at 30°C for 7 days (Ye et al., 2020b). After fermentation, a small amount of the solid agent was inoculated onto VY/2 medium to check for contamination and myxobacterial survival. One gram of the solid agent was suspended in 9 mL of sterile water, sonicated for 5 min (950 W, 30% output power, 3 s pulse on, 10 s pulse off), and serially diluted and plated to count colonies and calculate the myxobacterial population in the solid agent, which reached 10^6^ CFU/g.

*Verticillium dahlia* was inoculated onto PDA plates and incubated at 26°C for 7 days. Four mycelial plugs (*d* = 5 mm) were taken from the edge of the *V. dahliae* colonies using a sterile agar punch and inoculated into 200 mL of Czapek-Dox broth. The cultures were incubated at 26°C and 200 rpm for 6 days, filtered through four layers of sterile gauze, and diluted with sterile water to prepare a spore suspension of approximately 1.0 × 10^8^ spores/mL (Bai et al., 2022).

#### 2.7.2 Root drenching with HM-E fermentation broth

Cotton seedlings were prepared according to the method of Wei et al. (2019). Briefly, the unsterilized nutrient soil (composed of coconut coir, peat, carbonized rice husk, and perlite) was mixed with vermiculite at a 4:1 ratio and placed into plastic pots (40 cm × 20 cm × 15 cm). Cotton seeds were surface-sterilized with 1% sodium hypochlorite for 5 min, rinsed several times with sterile water, and germinated at room temperature. Once the radicles emerged, the seeds were sown, with 10 seedlings retained per pot. This experiment included four treatments. HM-E + Vd: At the two-leaf stage, 5 mL of HM-E fermentation broth (OD₆₀₀ = 1.0) was applied to the rhizosphere of each cotton seedling using a pipette. Carbendazim + Vd: 5 mL/plant of a 500-fold dilution of carbendazim (Shandong Zouping Pesticide Co., Ltd., active ingredient ≥50%) was applied. LBS + Vd: 5 mL/plant of sterile LBS broth was applied. Mock: Healthy cotton plants without any treatment served as the control. Seven days post soil drench inoculation with HM-E fermentation broth, carbendazim, and sterile LBS medium, the lateral roots of the cotton seedlings were wounded with a sterile bamboo stick, and 5 mL of *V. dahliae* spore suspension (1.0 × 10^8^ spores/mL) was applied to the rhizosphere of each cotton seedling. Ten cotton seedlings were planted per pot, and three pots constituted one treatment, with three replicates per treatment. Pots were randomly arranged in the greenhouse to reduce variations in light and temperature.

#### 2.7.3 Inoculation with HM-E solid agent

This experiment included four treatments. SFH (solid fermentation of HM-E) + Vd: At sowing, 0.5 g of the HM-E solid agent was applied below each seed in the planting hole, covered with a thin layer of soil (1–2 cm), and then sown. SSFS (sterile solid fermentation substrate) + Vd: 0.5 g of sterile solid fermentation substrate was applied below each seed at sowing. Vd: Only 5 mL of *V. dahliae* spore suspension was applied. At the two-leaf stage, 5 mL of *V. dahliae* spore suspension (1.0 × 10^8^ spores/mL) was applied to each cotton seedling (inoculation method as described in Section 2.7.2). Mock: Healthy cotton plants without any treatment served as the control. Ten cotton seedlings were planted per pot, and three pots constituted one treatment, with three replicates per treatment.

#### 2.7.4 Investigation of agronomic traits and disease control efficacy

The treated cotton plants were grown in a greenhouse with a temperature of 25–32°C and a relative humidity above 60% under conventional management. Pots were randomly placed in the greenhouse to minimize the influence of light and temperature differences. At 35 days post-*V. dahliae* inoculation, the vascular bundle discoloration of the cotton plants in each treatment was examined, and the disease incidence was recorded. The disease index was investigated according to the five-grade disease rating scale for cotton Verticillium wilt (Xue et al., 2013). The control efficacy was calculated as follows: Disease index = [∑(Number of diseased plants at each grade × Grade value)/(Total number of investigated plants × Highest grade value)] × 100; Control efficacy (%) = (Disease index of control − Disease index of treatment)/Disease index of control × 100. Meanwhile, the plant height, stem diameter, primary root length, shoot fresh and dry weight, and root fresh and dry weight of cotton were measured for the SFH + Vd, SSFS + Vd, and Vd treatment groups.

### 2.8 Inhibitory activity of HM-E cell-free fermentation filtrate and volatile organic compounds (VOCs) on *Verticillium dahlia* mycelial growth and spore germination

The HM-E fermentation broth was centrifuged at 12,000 × *g* for 15 min at 4°C, and the supernatant was collected. The supernatant was then filter-sterilized using a 0.22 μm membrane filter to obtain the cell-free fermentation filtrate. The cell-free fermentation filtrate was added to VY/2 medium at a ratio of 40% when the medium had cooled to approximately 50°C and mixed thoroughly. Sterile LBS broth was added to VY/2 medium at a ratio of 40% as a control. A *V. dahliae* mycelial plug (5 mm in diameter) was inoculated in the center of each plate, and the plates were incubated at 26°C. After 7 days, the plates were photographed, and the diameters of the *V. dahliae* colonies were measured. Each treatment was replicated three times. The mycelial morphology was also observed under a microscope (Ni-U, Nikon). For the spore germination assay, 1 mL of HM-E cell-free fermentation filtrate was mixed with 1 mL of *V. dahliae* spore suspension (1.0 × 10^8^ spores/mL) and co-incubated at 26°C and 180 rpm for 36 h. A mixture of sterile LBS broth and *V. dahliae* spore suspension at the same volume ratio served as the control. Each treatment was performed in triplicate. The number of spores and germinated spores were counted using a hemocytometer at 12 h, 24 h, and 36 h. Spore lysis rate and spore germination inhibition rate were calculated according to Xue et al. (2013), and the spore morphology was observed under a microscope (Ni-U, Nikon). Spore lysis rate (%) = (Number of spores in control − Number of spores in treatment)/Number of spores in control × 100. Spore germination rate (%) = (Number of germinated spores/100) × 100; Spore germination inhibition rate (%) = (Spore germination rate in control − Spore germination rate in treatment)/Spore germination rate in control × 100.

The antifungal activity of VOCs produced by strain HM-E was determined according to the method of Wu et al. (2015), which investigated the biocidal effects of volatile organic compounds produced by *C. fuscus* HM-E against fungal phytopathogens. 100 μL of HM-E cell suspension (OD₆₀₀ = 1.0) was spread on VY/2 agar plates and incubated for 3 days. A *V. dahliae* mycelial plug (5 mm in diameter) was inoculated in the center of another PDA plate. The two plates were placed face-to-face and sealed with Parafilm. Sterile VY/2 plates served as controls. Each treatment was performed in triplicate. After incubation at 26°C for 10 days, the plates were photographed, and the colony diameters were measured. The mycelial morphology was also observed under a microscope (Ni-U, Nikon).

### 2.9 Antifungal activity of metabolites, lipopeptides, and proteins from strain HM-E

#### 2.9.1 Metabolite extraction

Strain HM-E was cultured in LBS broth at 30°C and 180 rpm for 72 h. One liter of supernatant was collected by centrifugation and filter-sterilized through a 0.22 μm membrane filter to obtain the cell-free fermentation filtrate. Secondary metabolites were then extracted from the fermentation filtrate using macroporous resin XAD-16 (Amberlite™ XAD16N, 20–60 mesh, J&K Scientific, Beijing, China) (Nett et al., 2006). The resin was collected and washed with methanol to elute the adsorbed metabolites. The combined methanol eluates were rotary evaporated at 35°C under reduced pressure at 40 rpm until dryness. The crude extract obtained from the methanol extraction was dissolved in 5 mL phosphate-buffered saline (PBS, 10 mM) and filter-sterilized through a 0.22 μm membrane filter. The crude extract was obtained at a concentration of 214 mg/mL. The extract was serially diluted to a concentration of 100 mg/mL for further use.

#### 2.9.2 Lipopeptide extraction

The pH of 1 L of filter-sterilized (0.22 μm) HM-E fermentation broth was adjusted to 2.0 using 6 M HCl. After static precipitation at 4°C for 12 h, the mixture was centrifuged at 12,000 × *g* for 30 min at 4°C. The pellet was dissolved in 5 mL of methanol and then evaporated to dryness under reduced pressure at 35°C and 40 rpm (Yi et al., 2024). Subsequently, theprecipitate was collected and dissolved in 5 mL PBS to obtain a crude lipopeptide extract, with 702 mg/mL. The extract was then diluted to a concentration of 500 mg/mL for further use.

#### 2.9.3 Protein extraction and fractionation

To prepare a crude protein extract, ammonium sulfate was gradually added to 1 L of HM-E fermentation supernatant until 100% saturation was reached. The precipitated proteins were collected by centrifugation, dissolved in PBS, and dialyzed to remove salts. The protein concentration, determined using a BCA Protein Assay Kit (BCAP-1-W, Suzhou KeMing Biotechnology Co., Ltd., China), was 6.475 mg/mL. For protein fractionation, the HM-E culture supernatant was subjected to ammonium sulfate precipitation at increasing saturation levels (0–20%, 20–40%, 40–60%, 60–80%, and 80–100%). The precipitated proteins were collected, dissolved in PBS, and dialyzed as described above (Li et al., 2019b).

#### 2.9.4 Antifungal activity assays

To evaluate the antifungal activity of the secondary metabolites, lipopeptides, and crude proteins, a *V. dahliae* mycelial plug (5 mm in diameter) was inoculated in the center of a PDA plate. Two Oxford cups were placed symmetrically 2 cm away from the edge of the mycelial plug. 100 μL of the crude secondary metabolite extract, crude lipopeptide extract, and crude protein extract were added to separate Oxford cups. The plates were incubated at 28°C for 7 days, and the *V. dahliae* colony diameter was measured (Xue et al., 2013). PBS served as a negative control. Each treatment was performed in triplicate. In addition, 1 mL of each extract (secondary metabolites, lipopeptides, and crude proteins) was mixed with an equal volume of *V. dahliae* spore suspension (1.0 × 10^8^ spores/mL) in test tubes. A mixture of PBS and *V. dahliae* spore suspension at the same volume ratio served as the control. The mixtures were incubated at 30°C and 180 rpm for 24 h, each treatment was replicated three times. The number of spores and germinated spores were counted using a hemocytometer to calculate the spore lysis rate and spore germination inhibition rate (calculated as described in Section 2.8). The morphology of *V. dahliae* mycelia and spores in each treatment was observed under a microscope (Ni-U, Nikon).

To evaluate the antifungal activity of the extracellular proteins produced by strain HM-E against different plant pathogenic fungi, the antifungal activity of the 60–80% ammonium sulfate-precipitated protein fraction against *V.pyri, V. dahliae, F. verticillioides, A. tenuissima, F. culmorum, F. oxysporum* f. sp. *vasinfectum*, and *R. solani* was determined using the Oxford cup method as described above. Each treatment was replicated three times.

### 2.10 Effects of strain HM-E and its extracellular crude protein on *Verticillium dahliae* cell wall and membrane integrity and reactive oxygen species (ROS)

Four 5 mm diameter *Verticillium dahliae* (Vd) mycelial plugs, obtained using a sterile cork borer, were inoculated into PDB liquid medium and incubated statically at 26°C for 3 days. Mycelia were collected by centrifugation at 10,000 rpm for 15 min and stored for further use.

For the co-culture system, 1 mL of HM-E cell suspension (OD_600_ = 1.0) (prepared as described in section 2.6) and 0.1 g of fresh Vd mycelia were inoculated into 25 mL of co-culture medium (comprising 15 mL of TPM buffer [10 mM Tris–HCl, 1 mM KH_2_PO_4_, 8 mM MgSO_4_·7H_2_O], 5 mL of LBS medium, and 5 mL of PDB medium). A control treatment consisted of 1 mL of sterile water. In a separate treatment, 1 mL of crude extracellular protein (6.5 mg/mL) and 0.1 g of fresh Vd mycelia were inoculated into the same co-culture system, with 1 mL of PBS buffer as a control. Each treatment was performed in triplicate.

The co-culture systems were incubated at 30°C with shaking at 180 rpm for 12 h. At 6 h and 12 h post-inoculation, mycelia were collected and placed in sterile 1.5 mL centrifuge tubes. The mycelia were gently washed 2–3 times with PBS buffer to remove the culture medium, excess water, and other impurities and then stored for further use.

Cell wall integrity was assessed using Calcofluor White Stain (CWS) (Cat. No. SL7204, Beijing Coolaber Science & Technology Co., Ltd., China, Calcofluor White M2R 1 g/L, Evans blue 0.5 g/L). Ten microliters of CWS and 10 μL of KOH (0.18 M) were added to a clean glass slide, and a small amount of collected mycelia was immersed in the droplets. A coverslip was placed on the slide, and the preparation was allowed to stand for 1 min (Jofre et al., 2018). Samples at each time point in each treatment were prepared in triplicate.

To assess cell membrane integrity, propidium iodide (PI) (Cat. No. R20285, Shanghai Yuanye Biotechnology Co., Ltd., China, 5 μg/mL) was used. A small amount of treated mycelia was added to 10 μL of PI staining solution and incubated at room temperature in the dark for 30 min. After treatment, the mycelia were washed 2–3 times with sterile water (Ye et al., 2020a). Samples at each time point in each treatment were prepared in triplicate.

To detect ROS, a small amount of treated mycelia was resuspended in 90 μL of PBS buffer, and 10 μL of 2′, 7′-dichlorofluorescin diacetate (DCFH-DA) (Cat. No. D6470, Beijing Solarbio Science & Technology Co., Ltd., China, 1 mg/mL) was added. The mixture was incubated at 28°C for 30 min (Ye et al., 2020a). Samples at each time point in each treatment were prepared in triplicate. All basic protocols in the experiments were performed according to the manufacturers’ instructions. Fluorescence was observed using an AXR confocal laser scanning microscope (CLSM, Nikon, Japan).

### 2.11 Extracellular enzyme detection and substrate spectrum analysis of strain HM-E

To test strain HM-E’s ability to utilize different substrates, HM-E (OD₆₀₀ = 1.0) was spotted onto plates containing colloidal chitin, *β*-glucan, skim milk, starch, sodium carboxymethyl cellulose, and tributyrin. The plates were incubated at 30°C for 5 days. Each substrate was tested on three plates, and the experiment was repeated three times.

The ability of the enzymes to degrade various polysaccharides (chitin, pustulan, carboxymethyl cellulose, xylan, yeast glucan, mannan, starch, and *β*-1,3-glucan) was assessed using the DNS method on plates (Hu et al., 2008). Enzyme activity was quantified using a DNS Assay Kit (Beijing Solarbio Science & Technology Co., Ltd., China) following the manufacturer’s instructions. Each treatment was replicated three times.

Lipase activity was determined using *p*-nitrophenyl palmitate as a substrate, as described by (Zheng et al., 2011). A standard curve was generated using a series of diluted *p*-nitrophenol solutions. Inactivated crude enzyme solutions (100°C, 10 min) served as negative controls. Each treatment was replicated three times.

### 2.12 Statistical analysis

Statistical analysis was performed using standard analysis of variance (ANOVA) followed by Duncan’s multiple range test to determine significant differences between treatments. ANOVA was performed using SPSS 19.0 software. A significance level of *p* < 0.05 was considered statistically significant. Data analysis and graph generation were performed using GraphPad Prism 8 software.

## 3 Results and analysis

### 3.1 Characterization of strain HM-E

A myxobacterial strain, HM-E, was isolated using *Ea* as bait. On WCX medium, the fruiting bodies of this strain were mostly oval, single or clustered (Figures 1B,E), initially pale yellow, gradually darkening to bright brown (Figure 1B). On VY/2 plates, the bacterial film spread as an irregular thin layer with radial ridge-like protrusions (Figure 1C). The vegetative cells were thin rods, 3-10 μm in length (Figures 1D,F), and the myxospores were short rods (Figure 1D).

The growth characteristics and biochemical substrate utilization of strain HM-E were determined. The strain exhibited a growth temperature range of 16–40°C, a growth pH range of 6–9, and could not tolerate NaCl concentrations higher than 1.5%. It was able to hydrolyze starch, esculin, chitin, Tween 80, gelatin, and reduce nitrate, and produced urease. It did not produce catalase and oxidase. Strain HM-E exhibits resistance to 50 μg/mL ampicillin, 30 μg/mL tetracycline, and 30 μg/mL cefoxitin, but is susceptible to 50 μg/mL kanamycin and 10 μg/mL gentamicin. Some of the physiological and biochemical characteristics were consistent with those of the *C. fuscus* type strain DSM 2262 (Table 1).

**Table 1:** Comparison of characteristics of strain HM-E with different species of Cystobacter.

Substrates and conditions	Strains				
	Cystobacter minus DSM 14751^T^	Cystobacter violaceus DSM14727^T^	Cystobacter badius DSM 14723^T^	Cystobacter fuscus DSM 2262^T^	HM-E
Catalase	W+	W+	−	−	−
Oxidase	W+	W+	−	−	−
Tween 80	−	−	+	+	+
Aesculin	+	+	+	+	+
Urease	−	−	+	+	+
Chitin	+	+	ND	+	+
Ampicillin (50 μg ml^−1^)	+	−	+	+	+
Kanamycin (50 μg ml^−1^)	−	+	−	−	−
Temperature growth range (°C)	16–39	22–37	ND	18–37	16–40
pH tolerance	6–9	7–9	6–9	6–9	6–9
NaCl tolerance (%,w/v)	0–1.5	0–0.5	0–1	0–1.5	0–1.5

Data for strains other than HM-E are from Awal et al. (2017). W+, weakly positive; +, positive; −, negative; ND, not determined.

### 3.2 Molecular identification of strain HM-E

**Figure 2:**
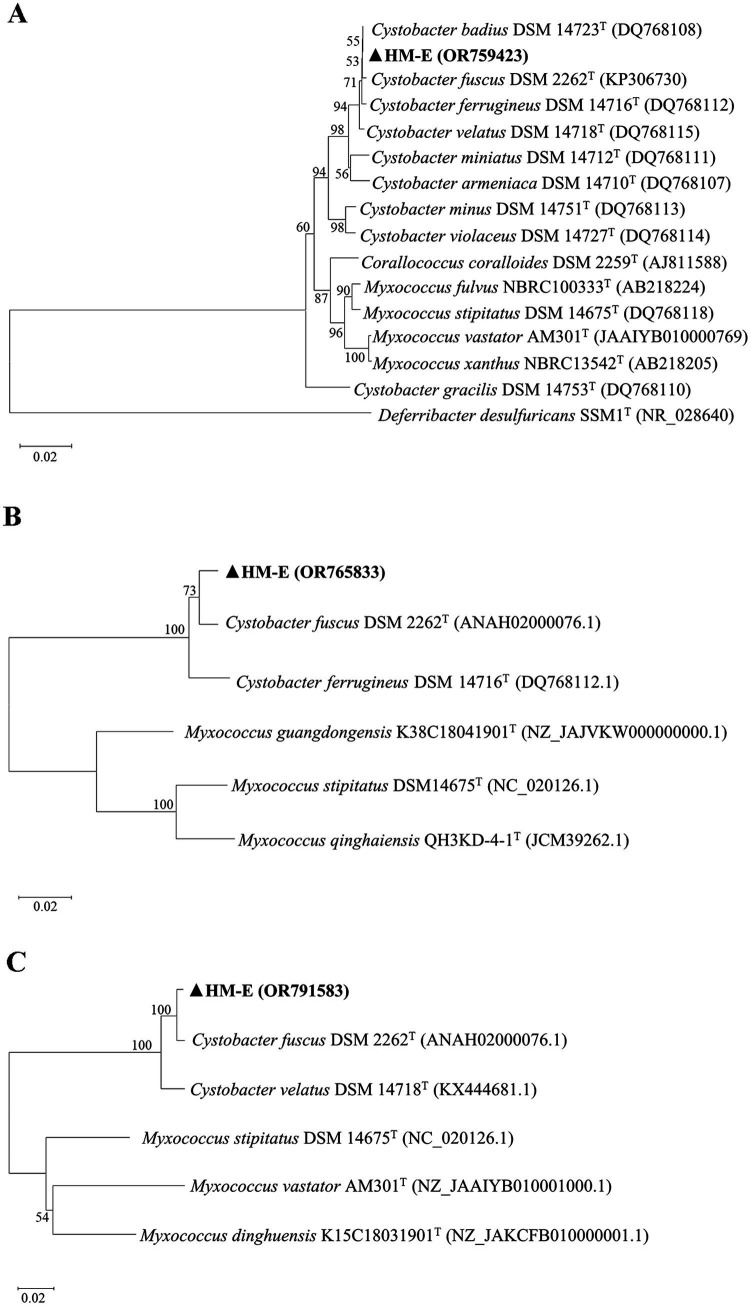
Molecular identification of strain HM-E. Phylogenetic trees based on 16S rRNA, *lepA*, and *gyrB* gene sequences were constructed using the Neighbor-Joining method in MEGA 11.0. **(A)** Phylogenetic tree of HM-E based on 16S rRNA sequences. **(B)** Phylogenetic tree of HM-E based on *lepA* sequences. **(C)** Phylogenetic tree of HM-E based on *gyrB* sequences.

Genomic DNA of strain HM-E was used as a template for PCR amplification and sequencing of the 16S rRNA, *lepA*, and *gyrB* genes. The obtained sequences were submitted to GenBank with accession numbers OR759423 (16S rRNA), OR765833 (*lepA*), and OR791583 (*gyrB*). In the phylogenetic trees constructed based on the 16S rRNA, *lepA*, and *gyrB* gene sequences, strain HM-E clustered with the *C. fuscus* type strain DSM 2262^T^ (Figure 2). Based on the combined morphological characteristics, growth characteristics, physiological and biochemical characteristics, and multi-gene sequence analysis, strain HM-E was identified as *C. fuscus*.

### 3.3 Antifungal activity of strain HM-E against various plant pathogenic fungi

**Figure 3:**
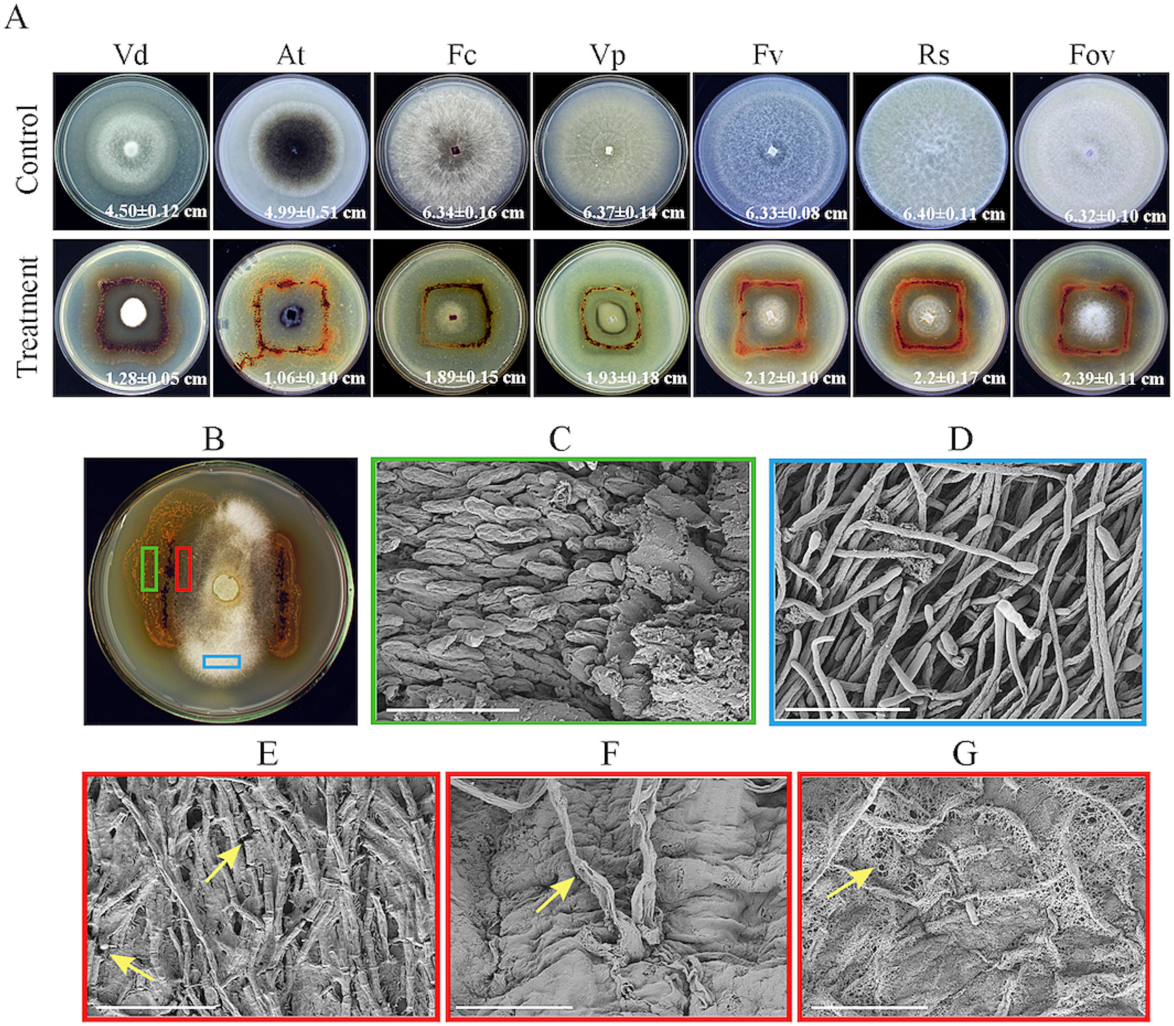
*In vitro* antagonism assays of strain HM-E against various phytopathogenic fungi and SEM observation of its predation on *Verticillium dahliae*. **(A)** Antagonistic activity of strain HM-E against various phytopathogenic fungi. Vd, *Verticillium dahliae*; At, *Alternaria tenuissima*; Fc, *Fusarium culmorum*; Vp, *Valsa mali* var. *pyri*; Fv, *Fusarium verticillioides*; Rs, *Rhizoctonia solani*; Fov, *Fusarium oxysporum* f. sp. *vasinfectum*. **(B)** Predation of strain HM-E on *Verticillium dahliae* on VY/2 agar, with marked green, red, and blue areas for subsequent sampling. **(C)** Magnified image of the green boxed area in (B), showing the formation and aggregation of numerous myxospores of strain HM-E prior to predation on *Verticillium dahliae*. Scale bar = 10 μm. **(D)** Normal hyphae of *Verticillium dahliae*. Scale bar = 40 μm. **(E)** Magnified image of the red boxed area in **(B)**, showing hyphal breakage of *Verticillium dahliae* after contact with strain HM-E. Yellow arrows indicate the breakage points. Scale bar = 40 μm. **(F)** Magnified image of the red boxed area in **(B)**, showing hyphal deformation and perforation of *Verticillium dahliae* after contact with strain HM-E. Yellow arrows indicate the perforation points. Scale bar = 10 μm. **(G)** Envelopment of *Verticillium dahliae* hyphae by strain HM-E. Yellow arrows indicate the reticular extracellular matrix secreted by strain HM-E. Scale bar = 20 μm.

Plate confrontation assays revealed that strain HM-E exhibited good antifungal activity against *V. dahliae*, *A. tenuissima*, *F. culmorum*, *V. pyri*, *F. verticillioides*, *R. solani*, and *F. oxysporum* f. sp. *vasinfectum* (Figure 3A), with particularly strong activity against *V. dahliae*. Observations showed that strain HM-E could spread along the hyphae, causing them to collapse and become sparse, and occupying a wider area, demonstrating obvious predation (Figure 3B).

Further scanning electron microscopy (SEM) analysis revealed that the vegetative cells of strain HM-E could move regularly along the *V. dahliae* hyphae, adhere to them, and cause extensive hyphal breakage (Figure 3E), malformation, and perforation (Figure 3F), compare wtih normal hyphae (Figure 3D). Strain HM-E also produced a large amount of net-like metabolites that enveloped the *V. dahliae* hyphae (Figure 3G). While those not involved predation hyphae aggregated into fruiting body (Figure 3C).

### 3.4 Greenhouse biocontrol efficacy of strain HM-E against cotton Verticillium wilt

**Figure 4:**
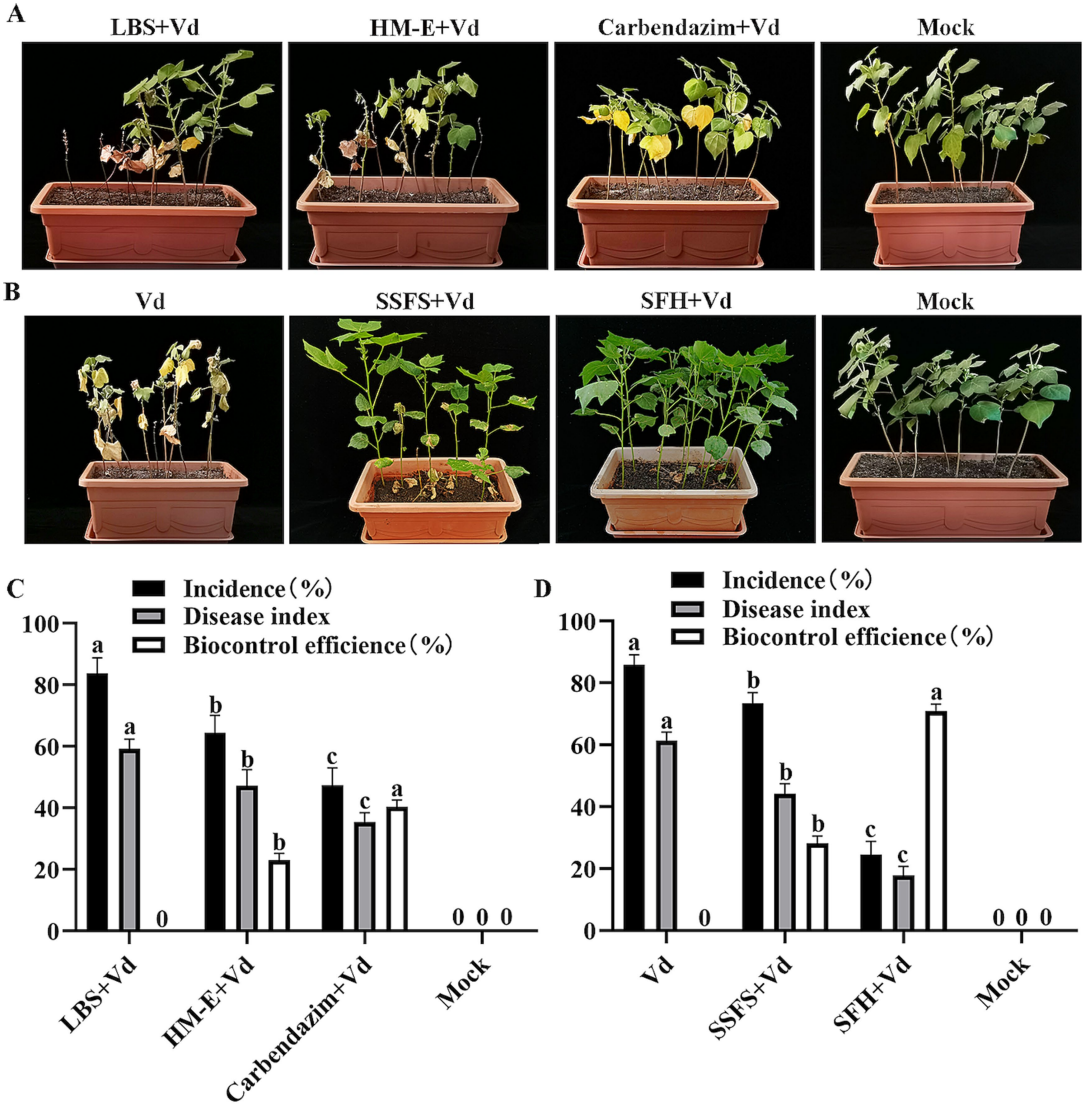
Evaluation of the biocontrol efficacy of strain HM-E against cotton Verticillium wilt at 35 days post-inoculation using two different application methods under greenhouse conditions. **(A)** Application of strain HM-E fermentation broth. LBS + Vd, sterile LBS medium inoculated with *Verticillium dahliae* spore suspension (1.0 × 10^8^ spores/mL); HM-E, strain HM-E fermentation broth inoculated with *Verticillium dahliae* spore suspension; Carbendazim, chemical fungicide Carbendazim inoculated with *Verticillium dahliae* spore suspension; mock, healthy cotton plants without *Verticillium dahliae* inoculation. **(B)** Application of HM-E solid agent. Vd, inoculation with *Verticillium dahliae* spore suspension only (1.0 × 10^8^ spores/mL); SSFS, sterile solid fermentation substrate; SFH, solid fermentation of HM-E; mock, healthy cotton plants without *Verticillium dahliae* inoculation. **(C)** Disease incidence, disease index, and control efficacy of cotton plants treated with root drench application. **(D)** Disease incidence, disease index, and control efficacy of cotton plants treated with solid agent application. Error bars represent standard deviations (± SD) of three independent replicates. Statistical comparisons were performed using Duncan’s multiple range test (*p* < 0.05). Groups marked with the same letter are not significantly different, while groups marked with different letters show statistically significant differences.

Root drenching experiments with HM-E fermentation broth showed that although the disease incidence and disease index of cotton seedlings in the HM-E fermentation broth treatment group were significantly lower than those in the control group (*p* < 0.05), the control efficacy was not ideal, only 23.01% (Figures 4A,C).

To improve the control efficacy, a myxobacterial solid agent was prepared using sterilized white star flower chafer frass as the solid fermentation substrate. The results showed that inoculation with the HM-E solid agent significantly reduced the disease incidence and disease index of cotton Verticillium wilt compared with the *V. dahliae*-treated group (*p* < 0.05), with a control efficacy of up to 70.90%. Although the sterile solid fermentation substrate also reduced the disease index of cotton seedlings, the control efficacy was only 28.14% (Figures 4B,D).

The experiments also showed (Table 2) that the HM-E solid agent treatment significantly increased plant height, main root length, shoot fresh/dry weight, and root fresh weight compared with the control and sterile solid fermentation substrate treatments, suggesting that strain HM-E may have certain plant growth-promoting functions.

**Table 2:** Evaluation of agronomic traits of cotton plants treated with strain HM-E under greenhouse conditions.

Treatment	Height/cm	Stem diameter/cm	Main root/mm	Aboveground fresh weigh/g	Aboveground dry weight/g	Underground fresh weight/g	Underground dry weight/g
SFH + Vd	37.26 ± 0.65a	3.20 ± 0.19a	32.09 ± 1.27a	59.20 ± 2.44a	23.65 ± 1.05a	14.80 ± 0.64a	5.61 ± 0.91a
SSFS + Vd	27.65 ± 1.31b	3.32 ± 0.24a	28.67 ± 0.27b	46.16 ± 2.26b	15.01 ± 1.75b	12.58 ± 0.85b	4.38 ± 0.19a
Vd	23.34 ± 1.66b	2.18 ± 0.15b	20.67 ± 0.27c	35.58 ± 1.68 c	9.60 ± 1.09c	6.61 ± 0.46c	2.20 ± 1.31b

Agronomic traits of cotton plants in each treatment group were assessed 35 days after inoculation with a *Verticillium dahliae* spore suspension (1 × 10^8^ spores/mL). SFH, solid fermentation of HM-E treatment; SSFS, sterile solid fermentation substrate treatment; Vd, control treatment inoculated only with *Verticillium dahliae* spore suspension (1 × 10^8^ spores/mL). Error bars represent standard deviations (± SD) of three independent replicates. Statistical comparisons were performed using Duncan’s multiple range test (*p* < 0.05). Groups marked with the same letter are not significantly different, while groups marked with different letters show statistically significant differences.

### 3.5 Inhibitory activity of HM-E cell-free fermentation filtrate and VOCs against *Verticillium dahliae*

**Figure 5:**
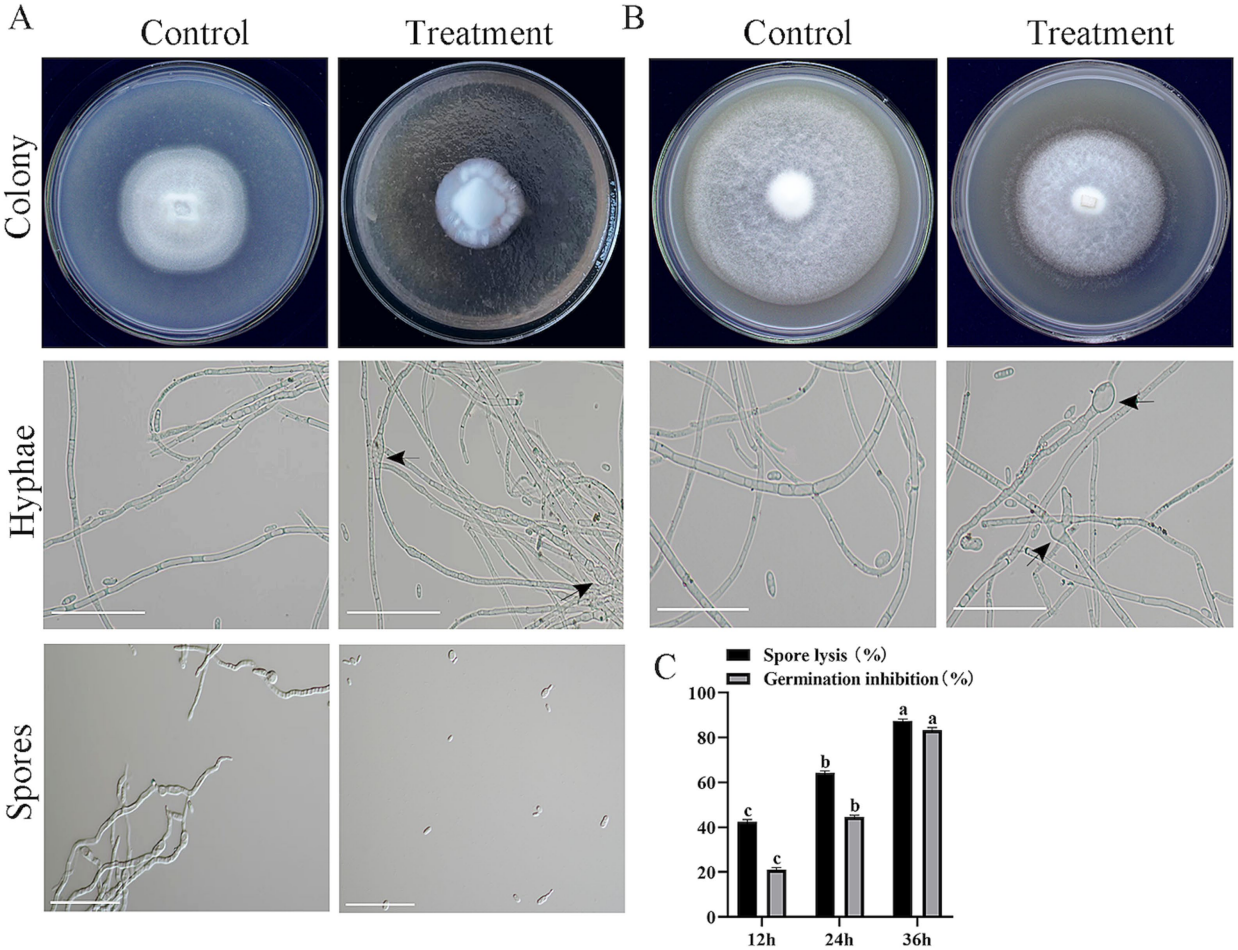
Inhibitory effects of strain HM-E sterile fermentation broth and volatile compounds on *Verticillium dahliae*. **(A)** Inhibitory effects of strain HM-E sterile fermentation broth on *Verticillium dahliae* colony growth, hyphal morphology, and spore germination observed on plates and under microscopy. Scale bar = 10 μm. Black arrows indicate hyphal swelling. **(B)** Inhibitory effects of strain HM-E volatile compounds on *Verticillium dahliae* colony growth and hyphal morphology observed on plates and under microscopy. Scale bar = 10 μm. Black arrows indicate hyphal swelling. **(C)** Lysis rate and spore germination inhibition rate of *Verticillium dahliae* spores treated with strain HM-E sterile fermentation broth at different time points. Error bars represent standard deviations (± SD) of three independent replicates. Statistical comparisons were performed using Duncan’s multiple range test (*p* < 0.05). Groups marked with the same letter are not significantly different, while groups marked with different letters show statistically significant differences.

Compared with the control, the cell-free fermentation filtrate of strain HM-E significantly inhibited the mycelial growth of *V. dahliae*, and the growth of aerial mycelia was suppressed. Microscopic observation revealed that the *V. dahliae* hyphae treated with the HM-E cell-free fermentation filtrate exhibited swelling (Figure 5A). The cell-free fermentation filtrate also lysed spores and inhibited spore germination (Figure 5A). At 36 h of incubation, the spore lysis rate was 87.48%, and the spore germination inhibition rate was 83.48% (Figure 5C).

In addition to the secreted metabolites in the medium, the activity of VOCs was also tested. Compared with the control group, the VOCs produced by strain HM-E significantly inhibited the mycelial growth of *V. dahliae* (Figure 5B). The hyphae at the edge of the *V. dahliae* colonies treated with VOCs were significantly sparse. Compared with the intact hyphae in the control group, the hyphae treated with VOCs exhibited swelling (Figure 5B).

### 3.6 Antimicrobial activity of extracellular secondary metabolites, lipopeptides, and proteins from strain HM-E

**Figure 6:**
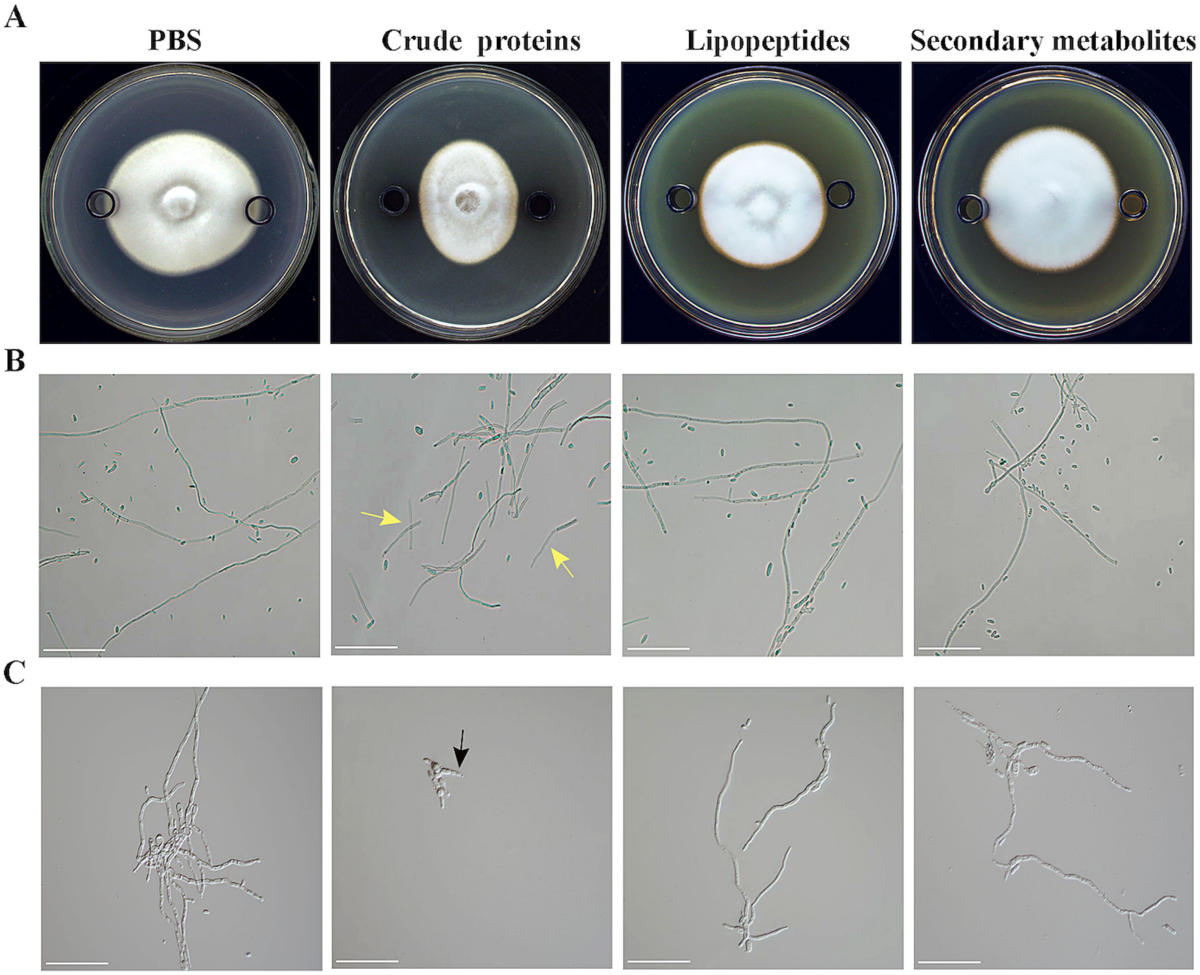
Antifungal activity of strain HM-E extracellular metabolites against *Verticillium dahliae*. **(A)** Co-cultivation of strain HM-E extracellular metabolites and *Verticillium dahliae* on PDA medium. **(B)** Microscopic observation of *Verticillium dahliae* hyphal growth after treatment with strain HM-E extracellular metabolites. Yellow arrows indicate hyphal breakage. Scale bar = 10 μm. **(C)** Microscopic observation of *Verticillium dahliae* spore germination after treatment with strain HM-E extracellular metabolites. Black arrows indicate inhibited germ tube emergence. Scale bar = 10 μm.

The Oxford cup method was used to determine the inhibitory activity of the extracellular secondary metabolites (100 mg/mL), lipopeptides (500 mg/mL), and crude proteins (6.475 mg/mL) of strain HM-E against *V. dahliae* mycelia and spores. The results showed that the extracellular secondary metabolites and lipopeptides of strain HM-E did not exhibit inhibitory activity against *V. dahliae* mycelial growth or spores (Figures 6A–C). However, the extracellular crude protein treatment showed obvious inhibitory activity, with significant collapse of the hyphae at the edge of the *V. dahliae* colonies (Figure 6A) and extensive hyphal breakage (Figure 6B). The number of *V. dahliae* spores was significantly reduced after treatment with the extracellular crude protein, and spore germ tube growth was inhibited (Figure 6C).

**Figure 7:**
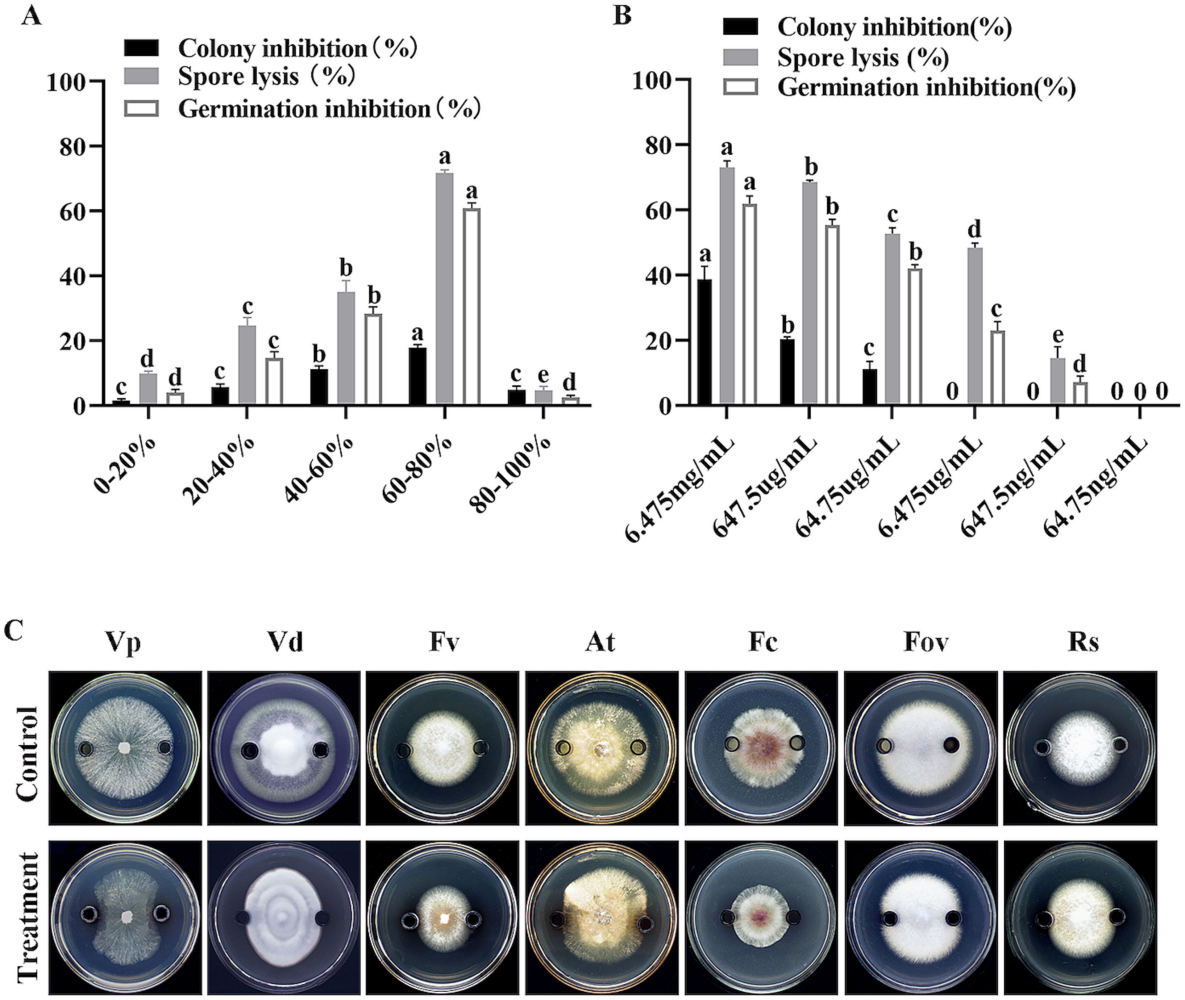
Effects of fractionated and serially diluted crude proteins from strain HM-E on *Verticillium dahliae* and various phytopathogenic fungi. **(A)** Effects of different protein fractions from strain HM-E on *Verticillium dahliae* growth inhibition, spore lysis rate, and spore germination inhibition rate. **(B)** Effects of serially diluted 60–80% protein fraction from strain HM-E on *Verticillium dahliae* hyphal growth inhibition, spore lysis rate, and spore germination inhibition rate. **(C)** Effects of the 60–80% crude protein fraction from strain HM-E on the growth of various phytopathogenic fungi. Vp, *Valsa mali* var. *pyri*; Vd, *Verticillium dahliae*; Fv, *Fusarium verticillioides*; At, *Alternaria tenuissima*; Fc, *Fusarium culmorum*; Fov, *Fusarium oxysporum* f. sp. *vasinfectum*; Rs, *Rhizoctonia solani*.

To further investigate the specific protein components responsible for this inhibitory activity, the extracellular proteins were fractionated by ammonium sulfate precipitation. The Oxford cup method was used to determine the inhibitory activity of different protein fractions. The results showed that the protein fraction obtained by 60–80% ammonium sulfate precipitation had the strongest inhibitory activity against *V. dahliae* mycelia, with an inhibition rate of 17.80% (Figure 7A). Co-culture of different protein fractions with *V. dahliae* spores also showed that the protein fraction obtained by 60–80% ammonium sulfate precipitation had the highest spore lysis rate and spore germination inhibition rate, which were 71.70% and 60.90%, respectively (Figure 7A). These results indicate that the inhibitory proteins are mainly present in the protein fraction precipitated by 60–80% ammonium sulfate. Serial dilution of the 60–80% ammonium sulfate-precipitated protein fraction revealed that the minimum inhibitory concentration (MIC) for mycelial growth was 64.75 μg/mL, and the MIC for spore germination was 647.5 ng/mL (Figure 7B).

Further experiments using the Oxford cup method showed that the 60–80% ammonium sulfate-precipitated protein fraction exhibited broad-spectrum antifungal activity against *V. dahliae*, *A. tenuissima*, *F. culmorum*, *V. pyri*, *F. verticillioides*, *R. solani*, and *F. oxysporum* f. sp. *vasinfectum*. The inhibitory activity was strongest against *V. phaseoli*, *V. dahliae*, and *F. verticillioides*, followed by *A. tenuissima* and *F. culmorum*. There was almost no antifungal activity observed against *F. oxysporum* and *R. solani* (Figure 7C).

### 3.7 Effects of extracellular crude protein from strain HM-E on *Verticillium dahliae* cell membrane and wall integrity and ROS production

**Figure 8:**
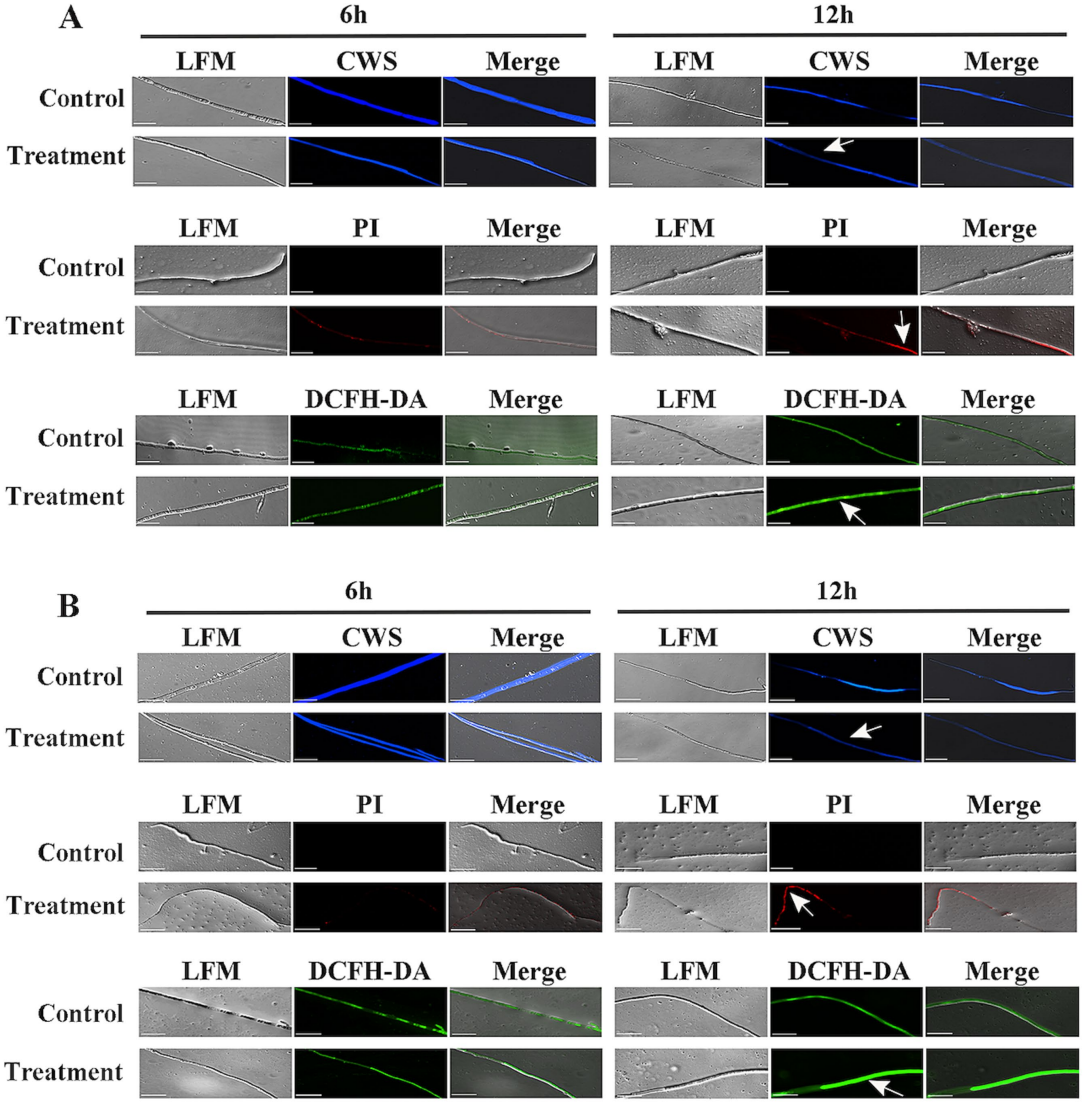
Analysis of reactive oxygen species (ROS) accumulation and cell wall/membrane integrity of *Verticillium dahliae* cells treated with strain HM-E and its crude proteins. **(A)** *Verticillium dahliae* hyphae co-cultured with the 60–80% fraction of strain HM-E crude proteins for 6 h and 12 h. **(B)** *Verticillium dahliae* hyphae co-cultured with strain HM-E for 6 h and 12 h. Cell wall integrity was assessed by CWS; cell membrane integrity was assessed by PI staining; and ROS accumulation was detected by DCFH-DA. Scale bar = 10 μm. LFM, light field microscopy; Merge, merged image of light and fluorescence fields showing hyphal morphology. White arrows indicate cell wall/membrane damage or ROS accumulation in *Verticillium dahliae* cells treated with strain HM-E or its crude proteins.

Calcofluor White Stain (CWS) is a non-specific fluorescent dye that binds to cellulose and chitin in fungal cell walls (Jofre et al., 2018). Compared with the control treatment, the blue fluorescence emitted from the hyphae gradually decreased with increasing co-incubation time of *V. dahliae* mycelia with the extracellular protein secreted by strain HM-E (Figure 8A), indicating that the integrity of the *V. dahliae* cell wall was disrupted by the extracellular protein secreted by strain HM-E.

Propidium iodide (PI) cannot penetrate intact cell membranes but can pass through damaged membranes and stain the nucleus (Kirchhoff and Cypionka, 2017). In this study, PI staining showed that red fluorescence began to accumulate in the hyphae 6 h after co-incubation of *V. dahliae* mycelia with the extracellular protein secreted by strain HM-E, and the red fluorescence gradually increased by 12 h (Figure 8A). These results indicate that the integrity of the *V. dahliae* cell membrane was disrupted by the extracellular protein secreted by strain HM-E.

Reactive oxygen species (ROS) play an important role in cell life (Cadenas and Davies, 2000). It has been reported that low concentrations of ROS in cells act as messengers, but a burst of ROS can lead to apoptosis or cell death (Brosche et al., 2014). Therefore, the ROS content in the treated cells was measured. 2′,7′-Dichlorofluorescin diacetate (DCFH-DA) staining showed that intracellular ROS significantly accumulated 6 h after co-incubation of *V. dahliae* mycelia with the extracellular protein secreted by strain HM-E (Figure 8A).

Furthermore, co-incubation of the HM-E cell suspension with *V. dahliae* mycelia, followed by CWS, PI, and DCFH-DA staining, also showed disruption of the cell membrane and cell wall and a burst of ROS (Figure 8B). Taken together, these results suggest that the extracellular proteins secreted by strain HM-E play an important role in the antimicrobial process.

### 3.8 Enzyme activity of strain HM-E

**Figure 9:**
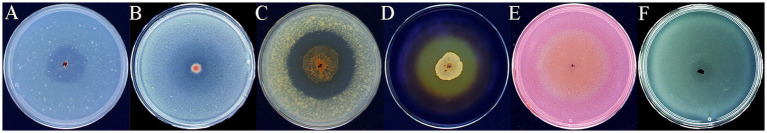
Hydrolytic activities of strain HM-E on various substrates. **(A)** Colloidal chitin, **(B)** *β*-glucan, **(C)** Skim milk, **(D)** Starch, **(E)** Sodium carboxymethyl cellulose, **(F)** Tributyrin.

The lytic activity of strain HM-E toward various substrates was investigated. The results demonstrated its ability to degrade colloidal chitin, *β*-glucan, skim milk, starch, sodium carboxymethyl cellulose, and tributyrin on plates. Among these, it showed the strongest activity on starch and skim milk plates (Figure 9).

The substrate spectrum of the 60–80% crude extracellular enzyme solution was further analyzed. By testing the hydrolytic activity of HM-E’s crude extracellular enzyme solution on various substrates, it was found that it could degrade starch, carboxymethyl cellulose, yeast glucan, xylan, chitin, *β*-1,3-glucan, mannan, and *p*-nitrophenyl palmitate, but not pustulan. Based on the chemical bonds present in these substrates, it is speculated that HM-E’s extracellular enzyme solution contains chitinase, glucanase, amylase, cellulase, and lipase. They did not exhibit hydrolytic activity toward pustulan, which contains *β*-1,6-glycosidic bonds. The specific activity was calculated according to the glucose standard curve (*y* = 0.1393x + 0.0006, R^2^ = 0.9905) (Table 3).

**Table 3:** The hydrolytic activity of crude enzyme extract (6.5 mg/mL) from strain HM-E on various substrates.

Substrates	Bond types	Enzyme activities (U/mL)
*p*-nitrophenyl palmitate	Ester linkage	477.53 ± 0.07
*β*-1,3-glucan	*β*-1,3-(Glucose)	63.75 ± 0.05
Yeast glucan	*β*-1,3-*β*-1,6-(Glucose)	63.71 ± 0.27
Xylan	*β*-1,4-(Xylopyranosyl)	63.70 ± 0.09
Starch	*α*-1,4-(Glucose); *α*-1,6-(Glucose)	5.90 ± 0.04
Carboxymethyl cellulose	*β*-1,4-(Glucose)	5.12 ± 0.06
Mannan	*α*-1,6-(Mannose)	1.60 ± 0.05
Chitin	*β*-1,4-N-Acetylaminoglycoside bond	1.25 ± 0.03
Pustulan	*β*-1,6-(Glucose)	0

Among the various enzymes tested, the crude enzyme solution exhibited the strongest hydrolytic activity toward *β*-1,3-glucan, reaching 63.75 ± 0.05 U/mL. The extracellular enzyme solution also contained lipase with ester bond hydrolytic activity. The specific activity was calculated based on the *p*-nitrophenol standard curve (*y* = 0.0398x − 0.0263, *R*^2^ = 0.993), and the hydrolytic activity toward *p*-nitrophenyl palmitate was the strongest, reaching 477.53 ± 0.07 U/mL (Table 3).

## 4 Discussion

Because *V. dahliae* can survive in soil for many years, crop rotation is difficult to implement. Coupled with a lack of effective resistant varieties and control agents, the damage caused by cotton Verticillium wilt has been increasing year by year, becoming a significant limiting factor for the healthy development of the cotton industry. In recent years, many beneficial microorganisms have been applied to the biocontrol of cotton Verticillium wilt with some success. Among them, biocontrol agents represented by *B. subtilis* and *Chaetomium* sp. have been developed into commercial microbial agents and applied for field control of cotton Verticillium wilt (Zhu et al., 2017). However, after entering open environments, biocontrol microorganisms are affected by environmental factors, plant rhizosphere regulation, and immune recognition during plant-microbe interactions, which can lead to difficulties in colonization and unstable control efficacy, thus limiting the practical application of biocontrol agents to some extent (Trdá et al., 2015; Zhalnina et al., 2018). Compared with reported biocontrol microorganisms, myxobacteria have broad-spectrum predatory and antibacterial activity, good soil colonization ability, and multiple biocontrol mechanisms, giving them natural advantages in biocontrol applications (Contreras-Moreno et al., 2024; Zhang et al., 2024). Studies have shown that myxobacteria exhibit good biocontrol effects against some plant bacterial (Dong et al., 2021; Han et al., 2024), fungal (Ye et al., 2020b), and oomycete diseases (Wu et al., 2021; Zhang et al., 2023b), and have good application potential, especially in the control of plant soilborne diseases (Iizuka et al., 2006).

In this study, a myxobacterial strain, HM-E, with broad-spectrum antifungal activity was isolated and screened. Based on its morphological, physiological, biochemical characteristics, and multi-locus sequence analysis, the strain was identified as *C.fuscus*. Plate confrontation assays showed that strain HM-E exhibited strong inhibitory activity against various plant pathogenic fungi, including *F. verticillioides*, *F. oxysporum* f. sp. *vasinfectum*, *R. solani*, and *V. dahliae*. On the plate surface, strain HM-E actively penetrated the *V. dahliae* colonies through swarming motility and caused extensive hyphal breakage. In greenhouse pot experiments, the strain showed good biocontrol efficacy against cotton Verticillium wilt, consistent with the plate assay results. The results indicate that strain HM-E is a potentially valuable biocontrol agent for cotton Verticillium wilt.

However, root-drenching inoculation with the HM-E fermentation broth did not exhibit ideal control efficacy, achieving only 23.01% control. Studies have found that myxobacterial solid agents exhibit significantly higher control efficacy than myxobacterial fermentation broth. For example, Ye et al. (2020b) compared the field biocontrol effects of *Corallococcus* sp. EGB broth and solid agent (prepared by solid-state fermentation of EGB) against cucumber Fusarium wilt and found that the EGB solid agent exhibited significantly better control than the EGB broth treatment. In field trials using a tobacco-rice rotation system, Yu et al. (2023) demonstrated that the biocontrol of plant pathogens was enhanced by combining myxobacteria with organic fertilizer (BF) compared to using myxobacteria alone (BS). In our preliminary experiments, we attempted to use rabbit and sheep manure as solid fermentation substrates for preparing myxobacterial solid agents. However, the pH of rabbit and sheep manure is higher than 8.0, which is not ideal for the colonization of myxobacteria, which prefer neutral or slightly alkaline conditions (The data are not shown). The *P. brevitarsis* frass used in this study for preparing the myxobacterial solid agent is produced through the fermentation of a mixture of cattle manure and cotton straw as feed, followed by digestion by *P. brevitarsis* larvae. Its pH is 7.0–7.5, which is more suitable for the growth of strain HM-E. The myxobacterial solid agent prepared using *P. brevitarsis* frass as the fermentation substrate exhibited good disease control efficacy as a basal fertilizer, achieving 70.90% control. The superior biocontrol efficacy of myxobacterial solid formulations prepared using insect frass and other organic materials, compared to myxobacterial fermentation broth, may be attributed to the following reasons: (a) In fermentation broth, myxobacteria primarily exist in the vegetative cell state, characterized by high metabolic activity but low stress tolerance and slow growth. When introduced into soil, these vegetative cells may struggle to rapidly adapt to the complex soil environment, making effective colonization difficult. (b) In contrast, myxobacterial solid formulations prepared using insect frass and other organic materials provide a nutrient-rich substrate that supports myxobacterial growth while also inducing the formation of stress-resistant fruiting bodies and myxospores. These structures enable myxobacteria to stably colonize the solid organic matrix. Even after entering the complex soil environment, the organic substrate serves as a protective niche, allowing myxobacteria to gradually adapt and establish in the soil. (c) Myxospores germinate slowly, progressively establishing a dominant population. In response to plant root exudates, myxobacteria undergo directed migration toward the rhizosphere, where they colonize (Ye et al., 2020b). During this migration, myxobacteria suppress plant diseases through predation and by modulating the soil microbial community structure (Yang et al., 2023).

In addition to disease control, the application of the HM-E solid agent significantly improved agronomic traits such as plant height and main root length compared with the *V. dahliae*-treated and sterile solid fermentation substrate treatments, suggesting that strain HM-E may have plant growth-promoting effects. Microorganisms with both antagonistic and growth-promoting activities are often more advantageous in the biocontrol of plant diseases, such as *Bacillus* spp., *Paenibacillus* spp., and *Streptomyces* spp. (Lapidot et al., 2015; Awla et al., 2017; Ben Abdallah et al., 2018). Their plant growth promotion is often attributed to the production of the plant hormone indole-3-acetic acid (IAA). However, based on previous results, strain HM-E does not have the ability to produce IAA. Therefore, its plant growth promotion may be due to its modulation of the cotton rhizosphere microbiome (Dawid, 2000).

Microorganisms that can produce secondary metabolites such as antibiotics are considered highly promising biocontrol agents, such as *Bacillus*, *Streptomyces*, and *Pseudomonas*. Myxobacteria can produce a rich variety of structurally novel secondary metabolites, some of which have strong inhibitory activity against plant pathogenic fungi and play an important role in controlling plant diseases (Iizuka et al., 2006). For example, Barbier et al. (2012). isolated the polyketide compound icumazole from *Sorangium cellulosum* So ce701, which has inhibitory effects on various fungi such as *Mucor hiemalis*, *Sclerotinia sclerotiorum*, *Botrytis cinerea*, and *Pythium debaryanum*. Wu et al. (2021) found that the secondary metabolites produced by *Myxococcus fulvus* B25-I-3 had strong inhibitory effects on the sexual and asexual reproduction of *Phytophthora infestans*.

This study found that the mycelial structure treated with the HM-E cell-free fermentation filtrate was disrupted, and a large number of spores were lysed and their germination was inhibited. Further extraction and concentration of secondary metabolites, lipopeptides, and extracellular crude proteins from the cell-free fermentation filtrate, followed by assays of their inhibitory activity, revealed that only the extracellular crude protein exhibited significant inhibitory activity. Therefore, we infer that the antifungal activity of strain HM-E against *V. dahliae* is almost not attributable to the extracellular secondary metabolites and lipopeptides secreted into the culture medium.

Volatile antifungal secondary metabolites (VOCs) produced by the myxobacterium *Corallococcus* sp. EGB can effectively inhibit *Penicillium* infection of oranges (Ye et al., 2020a), extending the postharvest shelf life of the fruit. In this study, we also found that the volatile metabolites (VOCs) produced by strain HM-E can inhibit the mycelial growth of *V. dahliae*.

In addition to secondary metabolites and VOCs, studies have shown that hydrolytic enzymes produced by antagonistic microorganisms play an important role in the biocontrol of plant pathogens (Srinon et al., 2006). For example, *β*-1,3-glucanase produced by *Streptomyces* exhibits strong antifungal activity against *Phytophthora capsici*, *R. solani*, *F.oxysporum*, *Fusarium crookwellense*, and *Paecilomyces* var*iotii* (Shi et al., 2010; Park et al., 2012). Furthermore, studies have reported that chitinases secreted by *Penicillium ochrochloron* and *Stenotrophomonas* can inhibit the growth of *Aspergillus niger* and *F. oxysporum* (Patil et al., 2013; Suma and Podile, 2013).

Myxobacteria can also produce various enzymes, including proteases, amylases, cellulases, lipases, chitinases, and xylanases which are the material basis for their predation (Munoz-Dorado et al., 2016). Zhou et al. obtained a *β*-1,3-glucanase (LamC27) from *Corallococcus* sp. EGB and found that it inhibited germ tube growth and appressorium formation of *Magnaporthe oryzae*, lysed hyphal cell walls, and induced ROS accumulation in spores and hyphae (Zhou et al., 2019). The *β*-1,6-glucanase (GluM), also from strain EGB, is the only reported outer membrane protein with glycoside hydrolase activity and has broad-spectrum antifungal activity, serving as a key factor in myxobacterial predation of fungi (Li et al., 2019b).

In this study, the extracellular crude protein secreted by strain HM-E exhibited certain inhibitory activity against various plant pathogenic fungi, including *V. dahliae*. Through fluorescence staining and microscopic observation, it was found that the extracellular crude protein secreted by strain HM-E could not only disrupt the cell wall and cell membrane of *V. dahliae* hyphae and cause intracellular ROS bursts but also lyse spores and inhibit spore germ tube growth. This is completely consistent with the results of co-culturing strain HM-E vegetative cells with *V. dahliae*. Therefore, we hypothesize that some extracellular enzymes secreted by strain HM-E play an important role in its predation of *V. dahliae* and its biocontrol of cotton Verticillium wilt.

Fungal cell walls are mainly composed of polysaccharides such as chitin, cellulose, mannan, and *β*-1,3-glucan. Microorganisms capable of producing enzymes that degrade these substances have potential for biocontrol development. For example, chitinase and *β*-1,3-glucanase produced by antagonistic microorganisms are key enzymes for degrading pathogenic fungal cell walls (Lorito et al., 1994). Wang et al. (2021) discovered a novel glycoside hydrolase family *β*-1,3-glucanase (AcGluA) in *Archangium* sp. AC19 and found that it has significant biocontrol effects on rice seedling defense. Li et al. (2019a) found that *Corallococcus* sp. EGB secretes a chitinolytic enzyme, CcCti1, which can hydrolyze colloidal chitin into N-acetylated chitohexaose (GlcNAc)₆, exhibiting high lytic activity against *M. oryzae*. Analysis of the hydrolytic activity of the HM-E crude enzyme extract against different substrates in this study revealed that strain HM-E possesses a rich array of glycoside hydrolase and lipase activities, indicating its potential for application in the biocontrol of fungal diseases.

Although this study confirmed through greenhouse pot experiments that strain HM-E has a good control effect on cotton Verticillium wilt, it has been reported that terrestrial environments with salinity higher than 1% are unfavorable for the growth of myxobacteria (Brenner and Staley, 2005). As Xinjiang’s soil is severely salinized, whether the screened myxobacteria can adapt to the saline-alkali soil environment in Xinjiang needs further verification and evaluation. The future application of myxobacterial products in Xinjiang cotton-growing areas will face challenges from various specific environmental conditions. Therefore, we are conducting field plot application tests and searching for cheaper and more readily available solid fermentation substrates.

## 5 Conclusion

This study successfully isolated and identified a myxobacterial strain, HM-E, exhibiting remarkable inhibitory activity against a range of important phytopathogenic fungi, including *V. dahliae*, *F. oxysporum* f. sp. *vasinfectum*, and *F. verticillioides*. Through morphological, biochemical, and molecular analyses, this strain was confirmed as *C. fuscus* HM-E. Crucially, rigorous greenhouse pot experiments demonstrated the outstanding biocontrol efficacy of a *C. fuscus* HM-E solid agent, formulated using *P. brevitarsis* frass as a carrier, against cotton Verticillium wilt, revealing its significant application potential. The extracellular enzymes secreted by *C. fuscus* HM-E, particularly peptidases, lipases, and glycoside hydrolases, are likely to play a key role in its antifungal mechanisms. This research provides a promising new biocontrol strategy and a valuable bioresource for combating the devastating cotton Verticillium wilt disease.

## Data Availability

The original contributions presented in the study are publicly available. This data can be found at: https://www.ncbi.nlm.nih.gov/search/all/?term=OR759423.
